# Comparison of Zika virus inactivation methods for reagent production and disinfection methods

**DOI:** 10.1016/j.jviromet.2020.114004

**Published:** 2020-10-21

**Authors:** Asiya S. Chida, Jason M. Goldstein, Joo Lee, Xiaoling Tang, Kanwar Bedi, Owen Herzegh, Jonathan L. Moon, David Petway, Dennis A. Bagarozzi, Laura J. Hughes

**Affiliations:** Reagent and Diagnostic Services Branch, Division of Scientific Resources, National Center for Emerging and Zoonotic Infectious Diseases, Centers for Disease Control and Prevention, Atlanta, GA, United States

**Keywords:** *Z*IKA virus, Inactivation, Cytopathic effect, Plaque assay, RNA stability, Antigenicityty

## Abstract

Zika virus (ZIKV) infection remains a public health concern necessitating demand for long-term virus production for diagnostic assays and R&D activities. Inactivated virus constitutes an important component of the Trioplex rRT-PCR assay and serological IgM assay (MAC-ELISA). The aim of our study is to establish standard methods of ZIKV inactivation while maintaining antigenicity and RNA integrity. We tested viral supernatants by four different inactivation methods: 1. Heat inactivation at 56 °C and 60 °C; 2. Gamma-Irradiation; 3. Chemical inactivation by Beta-propiolactone (BPL) and 4. Fast-acting commercial disinfecting agents. Effectivity was measured by cytopathic effect (CPE) and plaque assay. RNA stability and antigenicity were measured by RT-PCR and MAC-ELISA, respectively. Results: *Heat inactivation*: Low titer samples, incubated at 56 °C for 2 h, showed neither CPE or plaques compared to high titer supernatants that required 2.5 h. Inactivation occurred at 60 °C for 60 min with all virus titers. *Gamma irradiation:* Samples irradiated at ≥3 Mrad for low virus concentrations and ≥5Mrad for high virus titer completely inactivated virus. *Chemical Inactivation*: Neither CPE nor plaques were observed with ≥0.045 % BPL inactivation of ZIKV. *Disinfectant*: Treatment of viral supernatants with Micro-Chem Plus^™^, inactivated virus in 2 min, whereas, Ethanol (70 %) and STERIS Coverage^®^ Spray TB inactivated the virus in 5 min.

## 1. Introduction

Zika virus was first isolated in 1947 from a rhesus macaque (Dick et al., 1952) in Uganda and later from mosquitoes from the same region in 1948 (Faye et al., 2014). It belongs to *Flaviviridae* family and the genus flavivirus, which includes Japanese encephalitis virus, Yellow fever virus, West Nile virus and Dengue virus. It is enveloped and icosahedral with a non-segmented, single-stranded, positive-sense RNA genome. Zika virus is a vector-borne disease transmitted by *Aedes* species of mosquito, though there is evidence of person-to-person sexual transmission (Russell et al., 2017) Although ZIKV infection can cause an increased risk for development of Guillain Barre Syndrome in adults and *in utero* can lead to birth defects, such as microcephaly (Musso and Gubler, 2016; Pierson and Diamond, 2018; Saiz et al., 2017), most infections are asymptomatic or cause mild disease (Miguel et al., 2019). Zika virus has recently spread from the Pacific into the Americas when infection reached epidemic proportion in 2016. Based on complications associated from ZIKV, the World Health Organization declared a public health emergency of international concern (Bispo, 2016).

Though Zika virus infection is not currently a public health emergency, continuing spread of the virus and its vector requires increased testing, resulting in the demand for large-scale virus production to be used in diagnostic assays during pregnancy or those planning to become pregnant. ZIKV is an important component of the Trioplex Real Time RT-PCR kit and the IgM ELISA (MAC-ELISA).

There are several methods available to inactivate viruses for vaccines, diagnostic development, safe handling and disposal. For assay requirements, not all methods can be applied, based on the necessity for nucleic acids and native antigens. Additionally, each virus requires unique method development and validation. Currently, pasteurization, methylene blue treatment, low pH and solvent/detergent treatment methods are used to inactivate ZIKV in plasma and during manufacture of plasma -derived medicinal products (Wang et al., 2019; Blümel et al., 2017). In another study, Roth et al. described inactivation of a wide variety of enveloped and nonenveloped viruses by pasteurization with addition of stabilizers to be used in the manufacturing of therapeutic plasma proteins (Gröner et al., 2018). A comprehensive study of nine arboviruses from the three main arbovirus families (*Flaviviridae, Togaviridae*, and *Bunyaviridae*) for the development of a platform for production of inactivated arbovirus antigens in cell culture was performed by the Division of Vector Borne Diseases Arboviral Branch, at CDC (Goodman et al., 2014). The procedures were optimized for each viral species.

Due to the increased spread of infection, appropriate disinfectant for clinical and laboratory settings is necessary. In one report, inactivation and environmental stability of ZIKV using alcohol-based disinfectants, UV, heat treatment and changes in pH showed destruction of ZIKV, but these studies did not assess antigen or genomic stability (Müller et al., 2016).

The current study aims to investigate three inactivation methods for ZIKV for antigen production, nucleic acid extraction, and disinfection purposes. The effects of heat treatment, gamma irradiation, and BPL exposure were investigated on a range of virus titers. We sought to establish a standard method for ZIKV inactivation that retains the integrity of RNA and antigen. Inactivated virus constitutes an important component of the current Trioplex Real Time RT-PCR kit and MAC-ELISA. These tests are used to confirm active and recent infection, respectively. Additionally, ZIKV can be hazardous to basic researchers and to healthcare professionals that face exposure from clinical samples. These results provide information about minimum contact times for multiple disinfectants and standard procedures to inactivate ZIKV for production purposes.

## 2. Materials and methods

### 2.1. Cells and virus

Vero cells (ATCC CCL-81), used for the propagation and titration of ZIKV, were maintained in EMEM medium supplemented with 10 % fetal calf serum, L-glutamine and Penicillin/Streptomycin. ZIKV strain PRVABC59 (GenBank accession # KU501215) was obtained from the Centers for Disease Control and Prevention (Arboviral Diseases Branch, Division of Vector-borne Diseases.) (Kawai et al., 2019).

### 2.2. Propagation of Zika virus

Flasks of Vero cells at >95 % confluence were infected with ZIKV at 0.01 multiplicity of infection (MOI) in 10 mL of infection media (2 % FBS [Hyclone, GE healthcare, South Logan, Utah] in EMEM supplemented with L-glutamine, Penicillin/Streptomycin). Flasks were incubated for 1 h at 37 °C/5% CO_2_ with intermittent rocking. After 1 h, 50 mL of infection media was added to flasks for a total volume of 60 mL/flask and incubated for 7–8 days until optimal cytopathic effects (CPE) were observed. Culture supernatants were collected in 250 mL plastic bottles and the final concentration of FBS was brought to 20 %. Supernatants were then stored at −80 °C.

### 2.3. Cytopathic assay (CPE)

Vero cells were seeded in 6-well culture plates at 2 × 10^5^ cells/mL and incubated at 37 °C, 5% CO_2_, in saturated humidity. After reaching >95 % confluence, cells were incubated for 1 h with either 0.1 mL or 1 mL inactivated virus samples. Cells were washed 3 times with PBS. Virus inoculum was replaced with infection media, and the cells incubated at 37 °C for 7 days. The cells were examined and imaged for virus-induced cell changes and scored yes or no for CPE. Mock and live ZIKV were included in each assay as negative and positive controls, respectively.

### 2.4. Plaque assay

Plaque assays were performed according to previous reports (Goodman et al., 2015; Johnson et al., 2011; Basile et al., 2013; Beaty et al., 1995). Briefly, Vero cells were seeded in 6-well culture plates. Confluent cells (>95 % confluency) were incubated for 1 h with 0.1 mL of inactivated virus (10-fold serial dilutions of thawed virus supernatant were made starting from 10^−1^ to 10^−7^). A 3 mL overlay of 1 % agarose solution, 7.5 % sodium bicarbonate in Ye-Lah medium was added to the cells with virus after 1 h. Cells were incubated for 4 days at 37 °C. On day 5, a second overlay of 2 mL per well with overlay media incorporating 0.33 % Neutral red (Sigma Catalog # N2889) was added. On days 6, 7 and 8, the plates were monitored for plaques. Plates were imaged on the last day. Both live virus and mock-infected served as controls.

### 2.5. Heat inactivation

Cell culture supernatants (from 2.2) were thawed in a water bath at 42 °C for 30 min. Three virus concentration stocks were created: 1 × 10^5^ PFU/mL, 1 × 10^6^ PFU/mL and 1 × 10^7^ PFU/mL. Infection media (described above) was used for dilution of the samples. Aliquots (10 mL) of each titer were dispensed into 15 mL tubes (Falcon, 352097), aliquots (40 mL) of each concentration were dispensed into 50 mL tubes (Falcon, 352098) and aliquots (1 mL) of each concentration into 2 mL skirted, screw cap tubes (O-ring Sarstedt tubes, 72.694.006). Aliquots were incubated at 56 °C for 1.5, 2 and 2.5 h. Another set of aliquots were incubated at 60 °C for 10 min and 1 h. All samples were stored at −80 °C for later analysis.

### 2.6. BPL inactivation method

Thawed cell culture supernatants (from 2.2) (1 × 10^7^ PFU/mL) were dispensed into 5 mL aliquots in 15 mL tubes and treated with beta-propiolactone (BPL) (Sigma-Aldrich;) at final concentrations ranging from 0.01 % to 0.1 %. Samples were incubated at 4 °C for 3, 6 and 24 h with moderate rocking on a refrigerated rocker. Mock-treated control supernatants were subjected to the same conditions as the BPL-treated samples. 0.1 mL of 7.5 % sodium bicarbonate (Life Technologies) was added at 1 h and 6 h to prevent acidic pH levels. Samples were stored at −80 °C until later analysis.

### 2.7. Gamma irradiation procedure

Aliquots (5 mL) in 15 mL tubes were prepared at three virus titers: 1 × 10^5^ PFU/mL, 1 × 10^6^ PFU/mL and 1 × 10^7^ PFU/mL. All samples were placed on dry ice during gamma irradiation. For samples requiring longer exposures, dry ice was replenished every hour to prevent loss of viability due to temperature increase. Samples were exposed to 1–6 Mrad of gamma irradiation. Mock samples were left on dry ice without exposure to gamma irradiation. Samples were stored at −80 °C for later analysis.

### 2.8. Chemicals tested for viral disinfection

The following common decontamination chemicals were tested: 5 % Micro-chem Plus ^™^ (National Chemical Laboratories Cat # 0255), STERIS Coverage^®^ Spray TB ready to use disinfectant cleaner (n-alkyl dimethyl benzyl ammonium chloride, n-alkyl ethyl benzyl ammonium chloride, Isopronanol (Steris, Cat # 14–415-19) and 70 % Ethanol (Ricca laboratories, Catalog # 2546701). These chemicals were mixed with viral stocks (volume:volume) at 1:1 and 5:1 (disinfectant:virus) ratio. Incubation times were 1, 2, 5 and 10 min. After treatment, samples were stored at −80 °C until further analysis.

### 2.9. ZIKV real time-PCR assay

RNA was extracted from 200 μL of sample using the Magna Pure 96 system (Roche, catalog # 5195322001). All extractions were carried out according to the manufacturer’s recommendations. Real time PCR was performed immediately after extraction. Samples were tested for ZIKV using a single-reaction, multiplex rRT-PCR assay that detects ZIKA virus (ZIKV), Chikungunya (CHIKV), and Dengue (DENV) viruses as previously described (Trioplex Real-time RT-PCR assay, Centers for Disease Control and Prevention) (Santiago et al., 2018). Each run included nuclease-free water as a no-template control and RNA from live ZIKV as a positive control. Only the viral supernatants which showed no CPE nor plaques were tested in this assay.

### 2.10. Immunoglobulin M (IgM) antibody-capture enzyme-linked immunosorbent assay (Zika MAC-ELISA)

MAC-ELISA was performed as previously reported (Goodman et al., 2014). Only the viral supernatants which showed no CPE nor plaques were tested in this assay. Briefly, Immulon II HB Flat-bottomed 96 well plates were coated at 1:2000 with Anti-Human IgM (mu) Antibody (Kirkegaard and Perry Laboratories, Catalog # 5210–0157) overnight at 4 °C in carbonate/bicarbonate buffer. The wells were blocked with 5% nonfat milk in 1xPBS+0.5 % Tween-20 (Blocking buffer) for 30 min and wells washed with 1xPBS + 0.05 % Tween- 20, pH 7.2 using an automated plate washer 6.3 BioTek Auto Plate Washer (Model #, 405 LS or TS). Half of the plate was incubated with Flavivirus IgM positive control (ZV005, obtained from ADB/DVBD/CDC Ft Collins) and the other half with Flavivirus IgM negative control (AVR812, obtained from ADB/DVBD/CDC Ft Collins) for 1 h at 37 °C. Plates were washed and the wells incubated with samples overnight at 4 °C. Plates were washed and the wells incubated with Jackson-6B6C1-HRP (obtained from ADB/DVBD/CDC Ft Collins) [1/5000 dilution] in blocking buffer at 37 °C for 1 h. After washing the plates, Enhanced K-Blue TMB substrate (Neogen) was added to each well. After 10 min, stop solution was added and plates read at 450 nm on BioTek Microplate Reader (Gen5 software, version 3.03 secure).

### 2.11. Statistical analysis

Statistical analysis was performed using GraphPad Prism 8.0.1 software. All samples were tested in triplicate and the data range is represented by respective error bars. Ordinary one-way ANOVA was used to determine P value and R square. Comparison of live virus verses inactivation of each sample determined by Unpaired t-test, 2 tailed p value.

## 3. Results

### 3.1. Evaluation of inactivation

The inactivation of ZIKV by different methods (heat treatment, BPL treatment, gamma-irradiation) were evaluated by CPE and virus plaque assays. Vero cells were incubated with treated samples, and after 7 days, cells were assessed for morphological changes. Samples which showed complete inactivation (no CPE and no plaques) are marked as Y, and inactivation failed samples are marked as N in Table 1A, Table 1B–3. The fourth inactivation method, disinfectant treatments were analyzed by CPE alone (Table 4).

RNA stability was assessed using the Trioplex rRT-PCR assay. This method does not indicate intact genome, but rather that the region detected by primers included in this kit are detectable. Antigen stability was determined using the MAC-ELISA.

### 3.2. Heat inactivation

Exposure to 56 °C for 2.5 h in a 15 mL or 50 mL conical tube was sufficient to inactivate ZIKV up to a concentration of 1 × 10^7^ PFU/mL. Complete inactivation was not achieved in the 2 mL skirted tube even after 2.5 h of incubation (Table 1A). Incubation for 1 h at 60 °C was sufficient to inactivate all titers of virus in both types of conical tubes, while inactivation was still not observed in the 2 mL skirted tube (Table 1B). This suggest that either composition or thickness of the tube impacts heat treatment rather than sample volume.

**Fig. 1.**
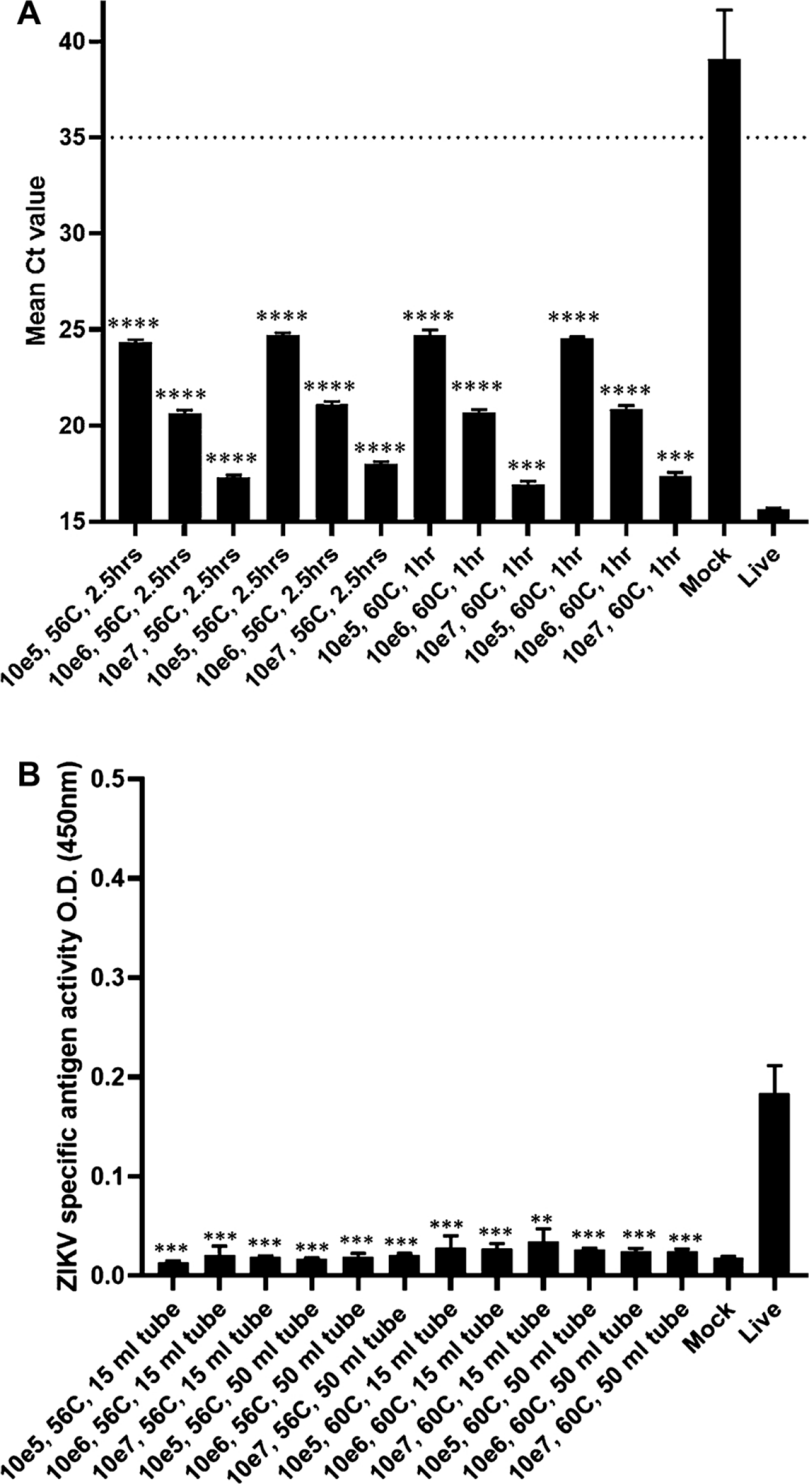
RNA and antigen stability after heat treatment. rRT-PCR was performed on all heat inactivated samples that demonstrated complete inactivation (A). All samples were run in triplicate and mean Ct value is represented with error bars on Y-axis. Ordinary one-way ANOVA gave P value <0.0001, R square = 0.9899. Ct value below 35 (cut-off line) is considered positive for ZIKV. For mock sample the value above 38 “may represent erratical amplification curves and is therefore considered negative” (Santiago et al., 2018). No template control (NTC) was undetermined. MAC-ELISA was performed on all heat inactivated samples that demonstrated complete inactivation (B). All samples were run in triplicate and ZIKV specific antigen activity is represented with error bars on Y axis as O.D. (450 nm). Ordinary one-way ANOVA gave P value <0.0001, R square = 0.9671. Inactivation of each sample compared with live virus analyzed by unpaired t-test, 2 tailed column analysis. p value < 0.01 (**); < 0.001 (***); <0.0001 (****).

RNA integrity was maintained in all heat-treated samples (Fig. 1A). However, MAC-ELISA results showed that antigen was no longer detectable after heat inactivation (Fig. 1B).

### 3.3. BPL inactivation

**Fig. 2.**
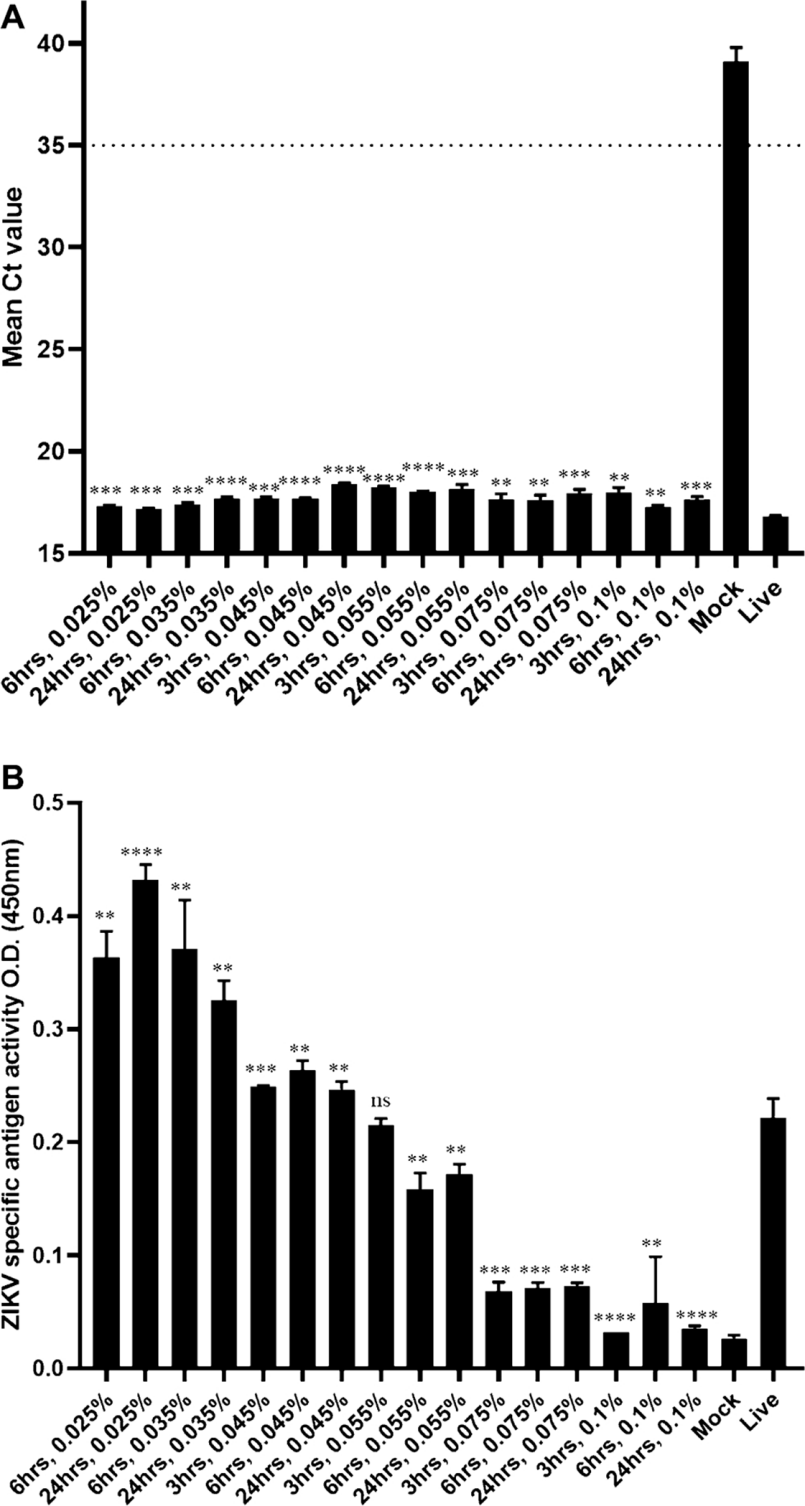
RNA and antigen stability after chemical inactivation with BPL. rRT-PCR was performed on BPL inactivated samples with BPL percentage and time points described on X-axis (A). Only samples that demonstrated complete inactivation were assessed for RNA. All samples were run in triplicate and mean Ct value is represented with error bars on Y-axis. Ct value below 35 (cut-off line) is considered positive for ZIKV. For mock sample the value above 38 “may represent erratical amplification curves and is therefore considered negative” (Santiago et al., 2018). No template control (NTC) was undetermined. Ordinary one-way ANOVA gave P value <0.0001, R square = 0.9988.MAC-ELISA performed on BPL inactivated samples with BPL percentage and time points described on X-axis (B). Samples that demonstrated complete inactivation were assessed by MAC-ELISA. All samples were run in triplicate and ZIKV specific antigen activity is represented with error bars on Y axis as O.D. (450 nm). Ordinary one-way ANOVA gave P value <0.0001, R square = 0.9870. Inactivation of each sample compared with live virus analyzed by unpaired t-test, 2 tailed column analysis. p value < 0.01 (**); < 0.001 (***); <0.0001 (****); ns = non-significant.

BPL titrations with concentrations ranging from 0.01 to 0.1 % were performed by incubating 5 mL of viral supernatants in a 15 mL conical tube for 3 h, 6 h and 24 h at 4 °C with mild rocking (Table 2). Cell toxicity resulting from BPL was tested in control experiments (data not shown). Therefore, to mitigate BPL toxicity, treated viral samples were diluted 1:10 prior to incubation with cells before CPE determination. A minimum of 0.025 % BPL-treatment for 6 h was required for inactivation of high titer virus (1 × 10^7^ PFU/mL). However, an increase to 0.045 % BPL is capable of inactivating virus at a titer of 1 × 10^7^ within 3 h without any appreciable loss in antigenicity and RNA stability. Increasing the concentration of BPL to 0.075 % and 0.1 % caused loss of antigenicity (Fig. 2B), although RNA was still detectable (Fig. 2A).

### 3.4. Gamma irradiation

**Fig. 3.**
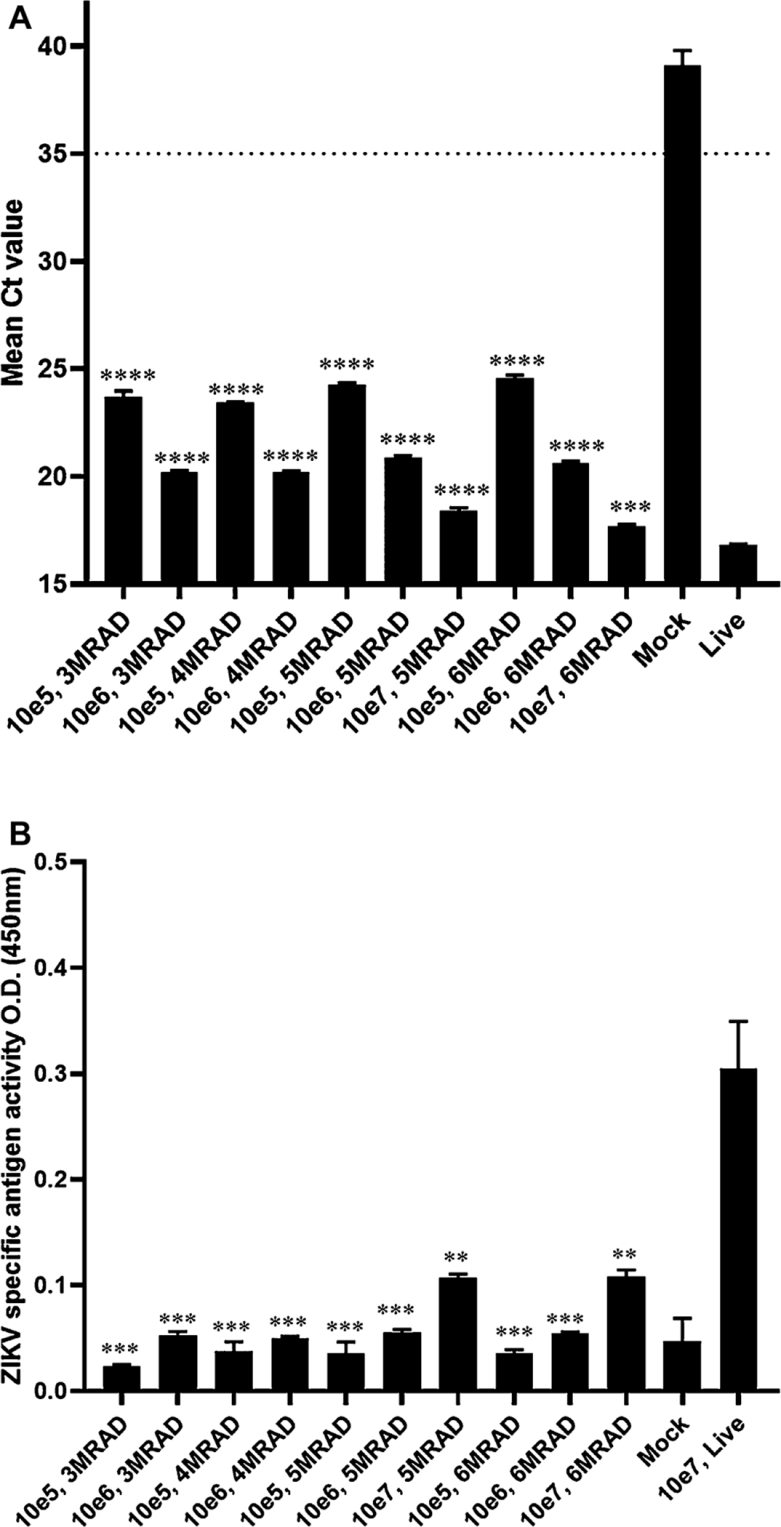
RNA and antigen stability after gamma irradiation. rRT-PCR was performed on gamma irradiated samples with different doses described on X-axis (A). Only samples that demonstrated complete inactivation were assessed for RNA. All samples were run in triplicate and mean Ct value is represented with error bars on Y-axis. Ct value below 35 (cut-off line) is considered positive for ZIKV. For mock sample the value above 38 “may represent erratical amplification curves and is therefore considered negative” (Santiago et al., 2018). No template control (NTC) was undetermined. Ordinary one-way ANOVA gave P value <0.0001, R square = 0.9989. MAC-ELISA performed was performed on gamma irradiated samples with different doses described on X-axis (B). Samples that demonstrated complete inactivation were assessed by MAC-ELISA. All samples were run in triplicate and ZIKV specific antigen activity is represented with error bars on Y axis as O.D. (450 nm). Ordinary one-way ANOVA gave P value <0.0001, R square = 0.9548. Inactivation of each sample compared with live virus analyzed by unpaired t-test, 2 tailed column analysis. p value < 0.01 (**); < 0.001 (***); <0.0001 (****).

Three different concentrations (1 × 10^5^ PFU/mL, 1 × 10^6^ PFU/mL and 1 × 10^7^ PFU/mL) of virus were treated with gamma irradiation doses ranging from 1 to 6 Mrad (Table 3). Control samples (untreated) remained on dry ice without any irradiation. The results demonstrate that at least 3 Mrad is required to inactivate at the lowest titer virus, but at least 5 Mrad is required for inactivation at 1 × 10^7^ PFU/mL. All doses of radiation resulted in decreases in antigenicity by 4–6-fold for all titers of virus compared to the corresponding live virus as determined by MAC-ELISA (Fig. 3B). However, RNA stability was preserved at all doses of gamma irradiation with all titers (Fig. 3A).

### 3.5. Treatment with chemical disinfectants

Viral supernatants (1 mL of 1 × 10^7^ PFU/mL) were treated with three types of disinfectant (70 % ethanol, 5 % Micro-Chem Plus^™^ and Steris Coverage^®^ Spray TB). This occurred at 1:1 or 5:1 (v:v) ratios of chemical to virus over 4 timepoints; 1 min, 2 min, 5 min and 10 min (Table 4). The results indicate that high virus titer sample mixed with 70 % ethanol (1:1) required 5 min exposure time, whereas 5% Micro-Chem and Steris with high titer virus required only 1 min. Increasing the disinfectant ratio to 5:1 for 70 % ethanol and Steris Coverage^®^ Spray TB required 2 min exposure time, whereas 5 % Micro-Chem Plus^™^ inactivated Zika virus within 1 min.

## 4. Discussion

The present study demonstrates a systematic evaluation of different inactivation methods for ZIKV. The aim was to compare the effects of these procedures on RNA stability and antigen integrity, which if maintained would enable production of large volumes of positive controls for current diagnostic assays. There are several methods to inactivate virus, but not all methods are appropriate for downstream applications (Sabbaghi et al., 2019; Zhang et al., 2019). Immunoassays for diagnostics or research may require a native protein without any structural modifications to protein antigens. The CDC Division of Vector-Borne Diseases (DVBD) Arboviral Diseases Branch (ADB) reference laboratory produces antigens for immunoassays covering a wide array of arboviruses. They have described the development of a cell culture antigen production algorithm for nine arboviruses from the three main arbovirus families, *Flaviviridae, Togaviridae,* and *Bunyaviridae* (Goodman et al., 2014). In order to establish an inactivation method for Zika virus, the present study compared the effect of established inactivation procedures such as heat treatment, chemical inactivation by BPL and gamma irradiation methods.

Heat treatment at 56 °C for 2.5 h was required to inactivate virus titers up to 1 × 10^7^ PFU/mL in 15 mL and 50 mL conical tubes, whereas heat treatment at 60 °C for 1 h was sufficient to inactivate all titers of virus in both tube types. We attribute the longer time required for inactivation at 60 °C than seen in previous work to the differences in volume and tube composition (Müller et al., 2016). Surprisingly, heat treatment in 2 mL tubes at both temperatures failed to inactivate virus even after 2.5 h. We believe that material and thickness of the tube are two critical parameters for heat dispersement. After heat treatment, RNA stability was maintained in all samples as determined by rRT-PCR testing. However, antigen was no longer detectable by the MAC-ELISA. This indicates that heat denatures the conformational epitope of ZIKV envelope protein typically detected in the assay. Therefore, heat treatment is incompatible with the MAC-ELISA or other immunoassays dependent on native antigen reactivity. This method is the most inexpensive and requires no chemicals.

With regard to BPL treatment, the results indicate that low concentrations of BPL (0.01 %) failed to inactivate the virus after 24 h. However, higher concentrations of BPL (0.025 % and 0.035 %) inactivated the virus within 6 h. The ideal percentage of BPL for inactivation of Zika virus ranges between 0.025 % and 0.045 % with an incubation time greater than 6 h. Higher concentrations of BPL (0.075 % and 0.1 %) produced cell toxicity and reduced antigenicity. Overall, ZIKV samples treated with BPL maintain antigenicity and RNA stability. Therefore, this method is suitable for inactivation in assays when RNA and native antigen integrity is critical. This has been previously determined for the MAC-ELISA, which utilizes ZIKV samples inactivated with BPL.

The use of gamma irradiation shows that inactivation of low titer virus requires at least 3 Mrad, but a total of 5 Mrad is required to inactivate virus at 1 × 10^7^ PFU/mL. However, detection in the MAC-ELISA was diminished for all virus titers at all doses required to inactivate virus. RNA stability was maintained at all doses of gamma irradiation. This method has advantages over BPL in terms of pH stability and limited modification to antigen. Furthermore, this procedure occurs at very low temperatures thus limiting degradation of the virus particles. One major disadvantage is that this process is very expensive and requires a radiation facility with certified and dedicated staff.

The following disinfectants were used: 5 % Micro-chem Plus ^™^, STERIS Coverage^®^ Spray TB ready to use disinfectant cleaner, and 70 % Ethanol at 1:1 and 5:1 ratio (volume:volume, chemical:virus) to investigate disinfectants for inactivation of ZIKV in the shortest time possible. With a 1:1 ratio, 70 % ethanol required a 5-minute exposure time for high virus titer, while 5 % Micro-Chem Plus^™^ and Steris Coverage^®^ Spray TB required 2 min to inactivate the virus. With an increased ratio of chemical (5:1), 70 % ethanol and Steris Coverage^®^ Spray TB were capable of killing virus after a 2-minute exposure time, while 5 % Micro-Chem Plus^™^ inactivated ZIKV within a 1 min exposure time. This suggests that 5 % Micro-Chem Plus^™^ can be used in hospital and clinical settings for sanitization.

In summary, the results indicate that virus concentration and surface area and material of the tube are critical factors dictating the effectiveness of heat inactivation methods. For gamma irradiated samples virus concentration and radiation dose are important parameters in inactivating the virus. Essentially, 5 Mrad is required to inactivate the virus at titers of 1 × 10^7^ PFU/mL and for titers of 1 × 10^5^ PFU/mL, greater than 3 Mrad is required. BPL inactivation is more dependent on the percentage of BPL over time. Higher than 0.055 % BPL is toxic to cells. The ideal percentage of BPL to completely inactivate the virus at 4 °C is 0.035 % to 0.045 %. RNA integrity is maintained in all these methods. However, heat- inactivated and gamma irradiated samples lost antigen stability.

The current study determined Micro-Chem Plus^™^ to be the most efficient chemical in destroying virus infectivity in the shortest time period. This information will help in day-to-day handling of infectious samples in the research and public health laboratory settings.

## Figures and Tables

**Table 1A:** Heat inactivation at 56 °C.

Temp	Tube	Volume	Time	Pfu/mL	Inactivated (Y/N)

56 °C	2 mL	1 mL	1.5 h	1 × 10^5^	N
				1 × 10^6^	N
				1 × 10^7^	N
			2 h	1 × 10^5^	N
				1 × 10^6^	N
				1 × 10^7^	N
			2.5 h	1 × 10^5^	N
				1 × 10^6^	N
				1 × 10^7^	N
56 °C	15 mL	10 mL	1.5 h	1 × 10^5^	N
				1 × 10^6^	N
				1 × 10^7^	N
			2 h	1 × 10^5^	Y
				1 × 10^6^	N
				1 × 10^7^	N
			2.5 h	1 × 10^5^	Y
				1 × 10^6^	Y
				1 × 10^7^	Y
56 °C	50 mL	40 mL	1.5 h	1 × 10^5^	N
				1 × 10^6^	N
				1 × 10^7^	N
			2 h	1 × 10^5^	Y
				1 × 10^6^	N
				1 × 10^7^	N
			2.5 h	1 × 10^5^	Y
				1 × 10^6^	Y
				1 × 10^7^	Y

A and 1B The inactivation of ZIKV by heat treatment was performed at 56 °C (Table 1A) and 60 °C (Table 1B). For heat treatment,10 mL of sample was dispensed into 15 mL conical tubes, 40 mL sample into 50 mL conical tubes and 1 mL sample into 2 mL screw cap tubes. For each condition, three titers of viral supernatant were tested: 1 × 10^5^ PFU/mL, 1 × 10^6^ PFU/mL and 1 × 10^7^ PFU/mL. The tubes at 56 °C (Table 1A), were treated for 1.5–2.5 hours in 30 min increments. The tubes at 60 °C (Table 1B) were dispensed similarly and treated for 10 min and 1 h. Y: inactivation was successful (no CPE and no plaques); N: inactivation failed (positive for CPE or plaque formation).

**Table 1B:** Heat inactivation at 60 °C.

Temp	Tube	Volume	Time	Pfu/mL	Inactivated (Y/N)

60 °C	2 mL	1 mL	10 min	1 × 10^5^	N
				1 × 10^6^	N
				1 × 10^7^	N
			1 h	1 × 10^5^	N
				1 × 10^6^	N
				1 × 10^7^	N
60 °C	15 mL	10 mL	10 min	1 × 10^5^	N
				1 × 10^6^	N
				1 × 10^7^	N
			1 h	1 × 10^5^	Y
				1 × 10^6^	Y
				1 × 10^7^	Y
60 °C	50 mL	40 mL	10 min	1 × 10^5^	N
				1 × 10^6^	N
				1 × 10^7^	N
			1 h	1 × 10^5^	Y
				1 × 10^6^	Y
				1 × 10^7^	Y

**Table 2:** BPL chemical inactivation.

BPL %	Time (hrs)	Inactivated (Y/N)

0.01	3	N
	6	N
	24	N
0.025	3	N
	6	Y
	24	Y
0.035	3	N
	6	Y
	24	Y
0.045	3	Y
	6	Y
	24	Y
0.055	3	Y
	6	Y
	24	Y
0.075	3	Y
	6	Y
	24	Y
0.1	3	Y
	6	Y
	24	Y

Chemical inactivation of ZIKV by BPL Samples (1 × 10^7^ PFU/mL) were treated with different concentrations of BPL as shown in the table. Each concentration was tested at 3 time points (3 h, 6 h and 24 h). Inactivation was assessed by CPE and plaque assay. Y: inactivation was successful (no CPE and no plaques); N: inactivation failed (positive for CPE or plaque formation).

**Table 3:** Gamma Irradiation.

Mrad	PFU/mL	Inactivated (Y/N)

1	1 × 10^5^	N
	1 × 10^6^	N
	1 × 10^7^	N
2	1 × 10^5^	N
	1 × 10^6^	N
	1 × 10^7^	N
3	1 × 10^5^	Y
	1 × 10^6^	Y
	1 × 10^7^	N
4	1 × 10^5^	Y
	1 × 10^6^	Y
	1 × 10^7^	N
5	1 × 10^5^	Y
	1 × 10^6^	Y
	1 × 10^7^	Y
6	1 × 10^5^	Y
	1 × 10^6^	Y
	1 × 10^7^	Y

Inactivation of ZIKV by gamma irradiation of three virus titers: 1 × 10^5^ PFU/mL, 1 × 10^6^ PFU/mL and 1 × 10^7^ PFU/mL was screened at gamma irradiation doses between 1 Mrad and 6 Mrad. Y: inactivation was successful (no CPE and no plaques); N: inactivation failed (positive for CPE or plaque formation).

**Table 4:** Chemical Disinfectant.

Chemical	Ratio C:V	Time (min)	Inactivated (Y/N)

70 % Ethanol	1:1	1	N
	1:1	2	N
	1:1	5	Y
	1:1	10	Y
5 % Micro-Chem Plus^™^	1:1	1	N
	1:1	2	Y
	1:1	5	Y
	1:1	10	Y
Steris Coverage^®^ Spray TB	1:1	1	N
	1:1	2	Y
	1:1	5	Y
	1:1	10	Y
70 % Ethanol	5:1	1	N
	5:1	2	Y
	5:1	5	Y
	5:1	10	Y
5 % Micro-Chem Plus^™^	5:1	1	Y
	5:1	2	Y
	5:1	5	Y
	5:1	10	Y
Steris Coverage^®^ Spray TB	5:1	1	N
	5:1	2	Y
	5:1	5	Y
	5:1	10	Y

Chemical disinfection of ZIKV. Three chemical disinfectants (70 % Ethanol, 5% Micro-chem plus and Steris Coverage Spray TB) were tested at 2 ratios (Chemical volume: Virus volume) 1:1 and 5:1 and assessed for efficacy after 1, 2, 5 and 10 min. Inactivation was confirmed by CPE assay. Y: inactivation was successful (no CPE); N: inactivation failed (positive for CPE).

## Abbreviations:

CPE: Cytopathic effect
ZIKV: Zika virus
BPL: Beta-propiolactone
